# β-arrestin2/miR-155/GSK3β regulates transition of 5′-azacytizine-induced Sca-1-positive cells to cardiomyocytes

**DOI:** 10.1111/jcmm.12339

**Published:** 2014-06-26

**Authors:** Jing Zhao, Yimin Feng, Hui Yan, Yangchao Chen, Jinlan Wang, Balvin Chua, Charles Stuart, Deling Yin

**Affiliations:** a Institute of Developmental Biology, School of Life Science, Shandong UniversityJinan, China; b Department of Internal Medicine, College of Medicine, East Tennessee State UniversityJohnson City, TN, USA; c School of Biomedical Sciences, Faculty of Medicine, The Chinese University of Hong KongHong Kong, China; d Cecile Cox Quillen Laboratory of Geriatrics, College of Medicine, East Tennessee State UniversityJohnson City, TN, USA

**Keywords:** stem cell antigen-1, cardiac stem cells, β-arrestin2, MiR-155, GSK3β, cardiomyocytes

## Abstract

Stem-cell antigen 1–positive (Sca-1+) cardiac stem cells (CSCs), a vital kind of CSCs in humans, promote cardiac repair *in vivo* and can differentiate to cardiomyocytes with 5′-azacytizine treatment *in vitro*. However, the underlying molecular mechanisms are unknown. β-arrestin2 is an important scaffold protein and highly expressed in the heart. To explore the function of β-arrestin2 in Sca-1+ CSC differentiation, we used β-arrestin2–knockout mice and overexpression strategies. Real-time PCR revealed that β-arrestin2 promoted 5′-azacytizine-induced Sca-1+ CSC differentiation *in vitro*. Because the microRNA 155 (miR-155) may regulate β-arrestin2 expression, we detected its role and relationship with β-arrestin2 and glycogen synthase kinase 3 (GSK3β), another probable target of miR-155. Real-time PCR revealed that miR-155, inhibited by β-arrestin2, impaired 5′-azacytizine-induced Sca-1+ CSC differentiation. On luciferase report assay, miR-155 could inhibit the activity of β-arrestin2 and GSK3β, which suggests a loop pathway between miR-155 and β-arrestin2. Furthermore, β-arrestin2-knockout inhibited the activity of GSK3β. Akt, the upstream inhibitor of GSK3β, was inhibited in β-arrestin2-Knockout mice, so the activity of GSK3β was regulated by β-arrestin2 not Akt. We transplanted Sca-1+ CSCs from β-arrestin2-knockout mice to mice with myocardial infarction and found similar protective functions as in wild-type mice but impaired arterial elastance. Furthermore, low level of β-arrestin2 agreed with decreased phosphorylation of AKT and increased phophorylation of GSK3β, similar to *in vitro* findings. The β-arrestin2/miR-155/GSK3β pathway may be a new mechanism with implications for treatment of heart disease.

## Introduction

Acute myocardial infarction, characterized by the irreversible necrosis of cardiac cells, causes a significant number of deaths every year. The clinical trials of stem cells transplantation have not been consistent because these cells either do not differentiate into cardiac cells or differentiate into only limited number of cardiac cells. More recently, direct differentiation of resident cardiac stem cells (CSCs) into cardiomyocytes has given new hope for myocardial regeneration [1–3]. However, the mechanisms of CSCs differentiation into cardiomyocytes are little known.

Several kinds of resident CSCs, including stem-cell antigen 1–positive (Sca-1+), c-kit+ and side-population cells, have been identified in adult hearts [2,4]. Transplantation of Sca-1+ into the infarcted area of hearts promotes cardiac repair [5,6], which indicates a key role of Sca-1+ resident CSCs in CSC differentiation and therapy. Recently, Sca-1+ cells were found to different into cardiomyocytes after treatment with 5-azacytidine (5aza) *in vitro* [7,8], this model helps in exploring the underlying mechanisms of Sca-1+ cell differentiation into cardiomyocytes.

β-arrestins, abundantly expressed in cardiac muscle, are well-known negative regulators of G-protein-coupled receptor signalling and function as scaffold proteins to modulate G-protein-independent signal cascades. β-arrestins consist of two proteins: β-arrestin1 and β-arrestin2 (Arrb2). The expression of Arrb2 is induced in the failing heart [9], and recent studies point to the beneficial role Arrb2 plays in the heart [10]. However, the direct function and the mechanism of Arrb2 mediated Sca-1+ CSC differentiation is not known yet.

MicroRNAs (miRNAs) are small 20- to 24-nt non-coding RNAs found in diverse organisms. They have a broad impact on gene expression *via* translational repression or post-transcriptional suppression [11]. TargetScan analysis showed that many miRNAs might regulate Arrb2. MiR-155 is a probable miRNA regulating Arrb2. Furthermore, miR-155 was greatly downregulated in a myocardial infarction model [12,13], so miR-155 might have a protective function in cardiac injury. However, whether miR-155 participates in Arrb2–regulated Sca-1+ cell differentiation is not clear.

In this study, we explored the mechanism of Arrb2 mediated Sca-1+ CSC differentiation, and found β-arrestin2/miR-155/GSK3β pathway regulates transition of 5′-azacytizine-induced Sca-1+ cells to cardiomyocytes, which might be a new target for the treatment of heart disease.

## Materials and methods

### Reagents

5aza and PKH2 green fluorescent cell linker kit were obtained from Sigma-Aldrich (St. Louis, MO, USA). Lipofectamine 2000, and SYBR GreenER were from Invitrogen (Grand Island, NY, USA). The MMLV reverse transcription system and dual luciferase reporter assay system were from Promega (Madison, MI, USA). TaqMan MicroRNA Assay, TaqMan MicroRNA Reverse Transcription kit, and TaqMan Universal PCR Master Mix were from Applied Biosystems (Foster, CA, USA). Antibodies, including total and phospho-GSK-3β (Ser 9), total and phospho-Akt (Ser 473), were from Cell Signaling Technology (Beverly, MA, USA). Biotinylated Sca-1 antibody was from BD Biosciences (San Jose, CA, USA). Antibodies for GAPDH and Arrb2 were from Santa Cruz Biotechnology (Santa Cruz, CA, USA). The cardiac troponin T (cTnT) antibody was from Abcam (Cambridge, UK). GSK-3β inhibitor SB216763 was from Tocris Bioscience (Bristol, UK).

### Animals

10–12 weeks Arrb2-KO mice on a C57BL/6 background were provided by Dr. Robert J. Lefkowitz (Duke University Medical Center, Durham, NC). Wild-type (WT) C57BL/6 male mice were obtained from the Jackson Laboratory (Bar Harbor, ME, USA). All mice were maintained in the Division of Laboratory Animal Resources at East Tennessee State University (ETSU), a facility accredited by the Association for the Assessment and Accreditation of Laboratory Animal Care International. Animal care and experimental protocols were approved by the ETSU Committee on Animal Care.

### Cells culture

Cardiac Sca-1+ cells were isolated by magnetic cell sorting from C57Bl/6 or Arrb2 knockout mice (10- to 12-week-old, C57Bl/6 background) with about 98% purity, as described previously [14]. Briefly, hearts from adult mice were treated with 0.1% collagenase for 30 min. followed by filtering through 80 μm mesh. To separate Sca-1+ cells, cells were incubated with biotinylated anti-Sca-1 antibody (BD Biosciences) for 15 min. on ice and washed with IMag buffer (consisting of PBS with 0.5% bovine serum albumin and 2 mM EDTA) followed by incubation with streptavidin-conjugated particles for 30 min. on ice. Newly isolated cardiac Sca-1+ were cultured on 1% gelatin-coated dishes with Iscove's modified Dulbecco's medium supplemented with 10% foetal bovine serum (FBS), 100 μg/ml penicillin, and 250 μg/ml streptomycin at 37°C in humid air with 5% CO_2_. The separated Sca-1+ CSCs were lack of the hematopoietic stem cell markers CD45 and CD34 (also a marker of endothelial progenitor cells) and hematopoietic transcription factors Lmo2, Gata2 and Tal [2]. At 1 day after seeding, cells were treated with 10 μM 5aza for the first 3 days; the medium was changed every 3 days. The dose and time of treatment with 5aza was reported previously [7,8]. Human HEK293T cells were purchased from American Type Culture Collection (USA).

### Cell transfection and plasmids

Sca-1+ cells (3.5 × 10^5^) in 350 μl gene pulse electroporation buffer with 40 μg/ml DNA were transferred into a 0.4-cm cuvette. After a pulse at 200 V, 250 μF, 1000 Ω, 10˝ with Bio-Rad MXcell (Bio-Rad, Hercules, CA, USA), cells were transferred to 1% gelatin-coated wells of a 24-well tissue culture plate containing 500 μl growth medium. Cells were incubated with Iscove's modified Dulbecco's medium supplemented with 10% FBS and 10 μM 5aza for the first 3 days, then normal culture medium. Arrb2 full-length and control vectors were generous gifts from Dr. Gang Pei (Shanghai Institutes for Biological Sciences).

### Real-time PCR (RT-PCR)

Total RNA was extracted and reverse-transcribed into cDNA. Quantified RT-PCR was involved use of SYBR GreenER on the Bio-Rad PCR instrument. PCR reaction conditions were according to the standard protocol. GAPDH was used as an endogenous control. All real-time PCR reactions were performed in triplicate, and relative quantification involved the ΔΔCt method (95% CI). All primer sets were subjected to a dissociation curve analysis and produced single peaks on a derivative plot of raw fluorescence. Primer sequences for MYH6, GATA4, cTnT and β-actin were as described [15].

### Western blot analysis

Total proteins were extracted by use of RIPA lysis buffer (Pierce Biotechnology, Rockford, IL, USA). Samples containing equal amounts of protein were separated by 8% SDS-PAGE and transferred onto Hybond ECL membranes (Amersham Pharmacia, Piscataway, NJ, USA), which were incubated overnight at 4°C with the appropriate primary antibodies (1:1000), then incubated 1 hr at RT with peroxidase-conjugated secondary antibodies (1:5000). Blots were exposed to the SuperSignal West Dura Extended Duration substrate (Pierce). Signals were quantified by scanning densitometry with the Bio-Image Analysis System (Bio-Rad).

### Luciferase reporter assay

HEK293T cells were seeded on 96-well plates the day before transfection in antibiotic-free medium. Cells were cotransfected with 60 ng miR-155 plasmid or control plasmid and 100 ng psicheck2 3′-UTR-WT (WT Arrb2 or GSK3β 3′-UTR) or psicheck2 3′-UTR-MUT (mutant miR-155 target site in Arrb2 or GSK3β 3′-UTR) by use of Lipofectamine 2000 (Invitrogen). After 48 hrs, cells were collected for luciferase assay with the Dual Luciferase Assay kit on a Modulus microplate. MiR-155 and control plasmids were generous gifts from Dr. Yangchao Chen (Chinese University of Hong Kong). Luciferase constructs were generated by Geneway Biotech Co. (Shanghai, China). Briefly, the entire 3′-UTR or 3′-UTR-MUT of Arrb2 and GSK-3β genes were cloned into pBluescript SK vector and then cloned into psicheck2 vector.

### Immunofluorescent staining

Cells or tissue slides were fixed with 3.7% formaldehyde in PBS for 20 min. at RT and stained with anti-cTnT antibody, then Alexa fluor 546-conjugated secondary antibody (Molecular Probes, Eugene, OR, USA). Cells or slides were examined by use of the Olympus IX70 microscope.

### Myocardial infarction-reperfusion (I/R) injury and cell delivery

Male mice were anesthetized with 5% isoflurance and maintained by inhalation of 1.5% isoflurance driven by 100% oxygen flow and ventilated by use of a rodent ventilator. Myocardial infarction was induced as described [16]. At 30 min. after left anterior descending ligation, 20 μl basal IMEM medium without cells (control group) or with 2 × 10^5^ Sca-1+ cells stained with PKH2 green fluorescent cell linker kit (cell injection group) were injected into the infarction and border zones of hearts by use of 29-gauge needles. After cell injection, hearts were reperfused for 1 hr, the chest was sutured with silk and all mice were allowed to recover. At 14 days after surgery, cardiac function was analysed. Every group has six mice and sham mice were as a control. At the end of the experiment, mice were killed, and hearts were collected for western blot analysis or were perfusion-fixed, embedded in paraffin, and cut transversely into 6–8 μm thick sections at the level of the papillary muscle. Sections were stained with anti-cTnT antibody and scanned.

### Cardiac functional analysis

Cardiac function was detected by use of the SPR-839 instrument (Millar Instruments, Houston, TX, USA) [16]. In anesthetized mice, systolic and diastolic arterial blood pressure was recorded by means of a microtip pressure transducer inserted into the right carotid artery. The catheter was then advanced into the left ventricle to measure cardiac functions in the closed-chest preparation. Then cardiac tissues were harvested for western blot and real-time PCR analyses.

### Statistical analysis

Data are reported as mean ± SEM and analysed by one-way anova followed by a Holm-Sidak *post hoc* analysis. Differences were considered statistically significant at *P* < 0.05.

## Results

### Arrb2 promoted 5aza-induced cardiac myocyte differentiation in CSCs

**Fig. 1:**
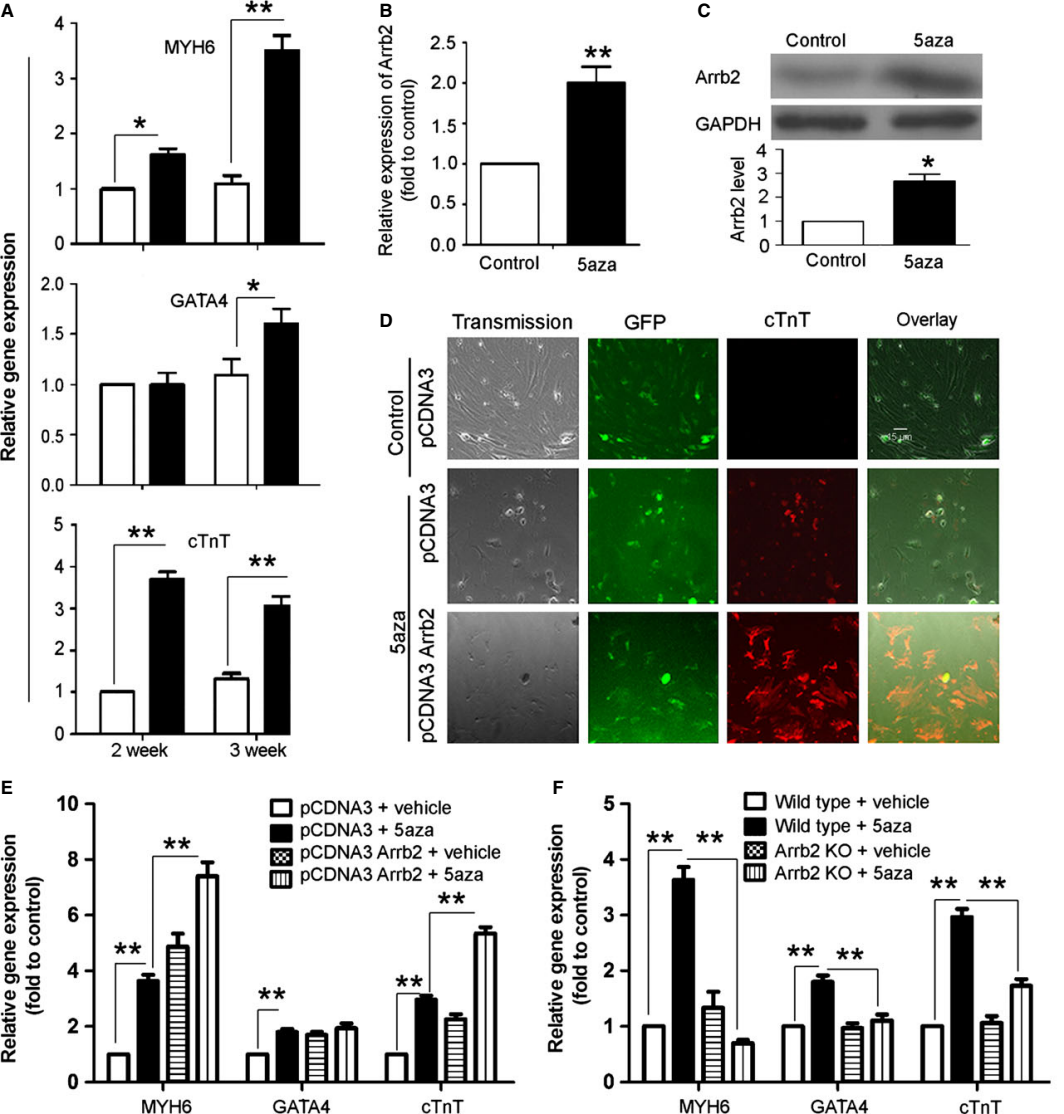
Effect of Arrb2 on 5′-azacytizine-induced differentiation of cardiac stem cells (CSCs) to cardiomyocytes. (**A**) Isolated Sca-1+ cells from wild-type (WT) mice were seeded 1 day before cells were treated with 5′-azacytizine (5aza) at 10 μM. After 3 days' treatment, cell culture medium was changed every 3 days for 2 and 3 weeks. Relative gene expression of cardiomyocyte markers including MYH6, GATA4, and cTnT were detected by RT-PCR. (**B** and **C**) isolated Sca-1+ cells from wild-type (WT) mice were treated with 5aza at 10 μM for 3 weeks. Arrb2 expression was determined by RT-PCR (**B**) and western blot analysis (**C**). (**D** and **E**) Sca-1+ cells from WT mice were transfected with full-length Arrb2 or control vector. After 24 hrs, cells were treated with 5aza as in **A**; the level of cTnT was detected by fluorescence assay (**D**) and the expression of MYH6, GATA4 and cTnT by RT-PCR (**E**). (**D**) It shows phase-contrast (transmission) and fluorescence images. GFP shows transfected cells; scale bar = 15 μm. (**F**) Sca-1+ cells from WT and Arrb2-knockout (KO) mice were treated with 5aza as in **A**. Real-time PCR analysis of the mRNA levels of MYH6, GATA4, and cTnT. Data are mean ± SEM of three experiments. *P < 0.05; **P < 0.01.

The expression of cardiomyocye markers showed that 5aza induced cardiac myocyte differentiation in Sca-1+ CSCs at 3 weeks (Fig. 1A). So this time-point was used in our current study. Arrb2 was up-regulated at both mRNA and protein levels at 3 weeks after 5aza treatment (Fig. 1B and C). Furthermore, Arrb2 overexpression could increase the mRNA expression of MYH6 and cTnT on RT-PCR and the level of cTnT on immunofluorescence assay (Fig. 1D and E), which suggested that Arrb2 promoted 5aza-induced Sca-1+ cell differentiation to cardiomyocytes.

To evaluate whether 5aza-induced Sca-1+ cell differentiation was through Arrb2, we used Sca-1+ cells from Arrb2-KO mice. 5aza could not up-regulate the expression of cardiac cell markers MYH6, GATA4 and cTnT in Sca-1+ cells from Arrb2-KO mice as compared with WT mice (Fig. 1F), which suggests that 5aza induced Sca-1+ cell differentiation *via* an Arrb2–dependent manner. Thus, we further determined the mechanisms responsible for Arrb2–dependent differentiation of 5aza-treated Sca-1+ cells.

### MiR-155 inhibited 5aza-induced myocardiac differentiation and was regulated by Arrb2

**Fig. 2:**
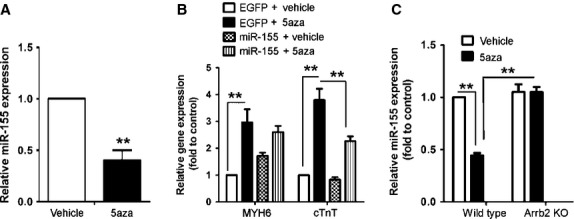
MiR-155 inhibits 5aza-induced differentiation of CSCs into cardiomyocytes through Arrb2. (**A**) RT-PCR analysis of the expression of miR-155 in Sca-1+ cells from WT mice treated as in Figure 1A. (**B**) Sca-1+ cells from WT mice were transfected with miR-155 plasmid or empty plasmid control. After 24 hrs, cells were treated with 5aza and the expression of MYH6 and cTnT was examined by RT-PCR. (**C**) Sca-1+ cells from WT and Arrb2-KO mice were treated with 5aza and miR-155 expression was examined as in **A**. Data are mean ± SEM of three experiments. **P < 0.01.

MiR-155 is a potential regulator for Arrb2 expression as suggested by analysis with Targetscan. We found miR-155 level decreased in CSCs after 3 weeks of 5aza treatment (Fig. 2A). To study the role of miR-155, we generated a construct expressing miR-155. Overexpression of miR-155 rescued the increased expression of the cardiac marker cTnT (Fig. 2B), so miR-155 inhibited the myocardiac differentiation.

To determine the relationship between miR-155 and Arrb2, we detected the changes in miR-155 level in Arrb2–transfected Sca-1+ CSCs by RT-PCR. Arrb2-KO inhibited the level of miR-155 in WT cells (Fig. 2C), which supports a relationship between Arrb2 and miR-155.

**Fig. 3:**
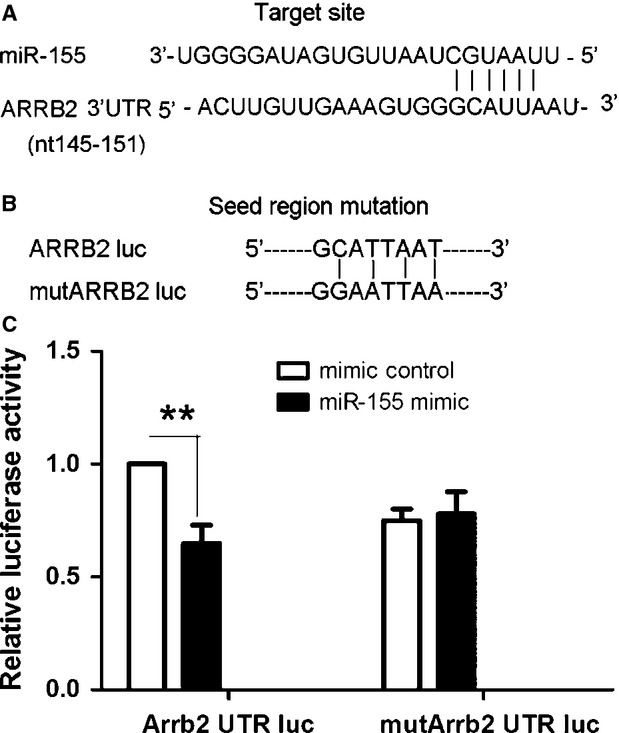
Arrb2 is a miR-155 target. (**A**) Sequence alignment of miR-155 and its target site in the 3′-UTR of Arrb2 (downloaded from http://www.targetscan.org). (**B**) The seed region of Arrb2 3′-UTR was mutated as indicated. (**C**) HEK293T cells were cotransfected with 60 ng miR-155 plasmid or empty EGFP plasmid control and 0.1 μg psicheck2 3′-UTR-WT (WT Arrb2) or psicheck2 3′-UTR-MUT (mutant miR-155 target site in Arrb2 3′-UTR). Cells were collected 48 hrs after transfection and analysed by dual luciferase reporter assay. The psicheck2 vector that provided the constitutive expression of Renilla luciferase was cotransfected as an internal control. Data are mean ± SEM of four experiments. **P < 0.01.

The potential target site for miR-155 interaction is at nucleotides 145-151 of the mouse Arrb2 3′-UTR as suggested by analysis with Targetscan (Fig. 3A). To test whether miR-155 could directly target the 3′-UTR of Arrb2 mRNA in a sequence-specific manner, we generated a luciferase construct harbouring a potential binding site for miR-155 and produced a mutant construct with potential target sites mutated (Fig. 3B). Luciferase activity decreased significantly in cells transfected with luc-β–arrestin2 on cotransfection with miR-155, with no significant difference in luciferase activity on cotransfection with the mutated construct and miR-155 (Fig. 3C). So miR-155 might target Arrb2 and inhibit its expression, but because Arrb2 will inhibit the expression of miR-155. Thus, there is a loop pathway between Arrb2 and miR-155 to maintain the function of Arrb2 promoting CSC differentiation.

### GSK3β is required for 5aza-mediated myocardiac differentiation and targeted by miR-155

**Fig. 4:**
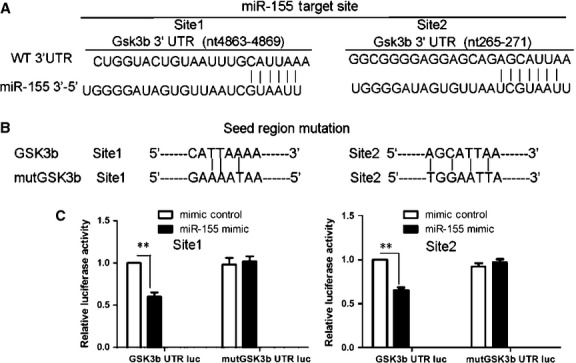
MiR-155 targets GSK3β. (**A**) Two possible GSK3β sites could be targeted by miR-155 as in Figure 3A. (**B**) The seed regions of GSK3β 3′-UTR were mutated as indicated. (**C**) Luciferase assay of psicheck2 3′-UTR-WT (WT GSK3β) or psicheck2 3′-UTR-MUT (mutant miR-155 target site in GSK3β 3′-UTR) and others measured as in Figure 3C. Data are mean ± SEM of four experiments. **P < 0.01.

To clarify the downstream molecule of miR-155, we analysed the function of GSK3β, another probable target of miR-155, determined *via* Targetscan, in 5aza-induced differentiation. Computational analysis indicated that miR-155 potentially targets mouse GSK3β at nucleotides 4863-4869 and 265-271 (Fig. 4A). We generated luciferase constructs harbouring two potential binding sites for miR-155 and produced a mutant construct with potential target sites mutated (Fig. 4B). Luciferase activity decreased significantly in luc-GSK3β–transfected cells on cotransfection with miR-155, with no significant difference on cotransfection with the mutated construct and miR-155 (Fig. 4C). So GSK3β is the target of miR-155.

**Fig. 5:**
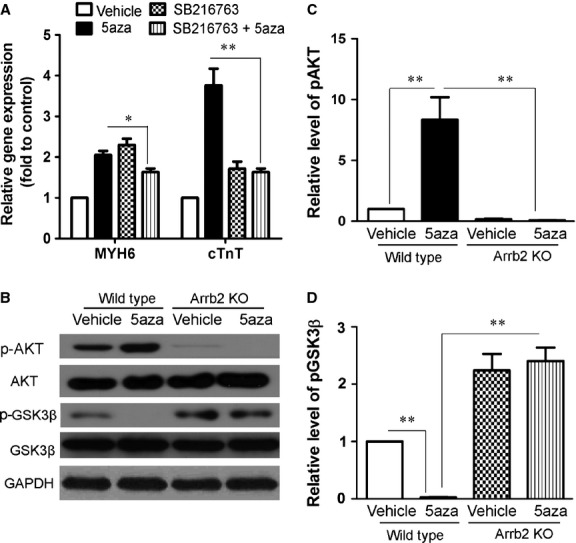
GSK3β is involved in 5aza-induced differentiation of CSCs to cardiomyocytes. (**A**) Sca-1+ cells from WT mice were treated with 5aza as in Figure 1A and incubated with or without SB216763 at 10 μM for the first 3 days. The mRNA expression of MYH6 and cTnT was analysed by RT-PCR analysis. (**B**) Sca-1+ cells from WT and Arrb2-KO mice were treated with 5aza as in Figure 1A. The expression of total and phosphorylated Akt (p-Akt), total and p-GSK3β were analysed by western blot. (**C**) Quantification of p-Akt levels shown in **B**. protein level were normalized to AKT. (**D**) Quantification of p-GSK3β levels shown in **B**. protein level were normalized to GSK3β. Data are mean ± SEM of four experiments. *P < 0.05; **P < 0.01.

To determine the function of GSK3β, we treated Sca-1+ cells with its inhibitor, SB216763, at 10 μM, together with 5aza for the first 3 days. The effective concentration was as described previously and in our preliminary experiment [17]. SB216763 could inhibit the 5aza-induced expression of cardiac markers (Fig. 5A), which suggested that the activity of GSK3β was required for 5aza-mediated myocardiocyte differentiation.

### 5aza promotes Sca-1+ cell transition to cardiomyocytes through an Arrb2/miR-155/GSK3β pathway

To analyse the signalling pathways involved in myocardiac differentiation, we examined the effect of Arrb2 on changes in GSK3β expression. 5aza inhibited the phosphorylation of GSK3β. However, Arrb2-KO in Sca-1+ cells promoted phosphorylation of GSK3β and inhibited its activity (Fig. 5B and D). Thus, Arrb2 participated in 5aza-induced CSC transition to cardiomyocytes by promoting GSK3β activation.

Akt is a well-known upstream inhibitor of GSK3β activation. To exclude the function of Akt on GSK3β activation, we detected changes in Akt activity. Phosphorylation of Akt was increased in 5aza-treated CSCs, and Arrb2-KO in Sca-1+ cells inhibited 5aza-induced activation of Akt. The mortality of the I/R model in mice is about 40%. Because all the change rules are in contrast to the changes in AKT as the inhibitor to GSK3β (Fig. 5B and C), we concluded that Arrb2 promoted the activation of GSK3β by inhibiting miR-155 but not Akt.

### Arrb2/miR-155/GSK3β pathway in CSC-mediated cardiac repair

**Fig. 6:**
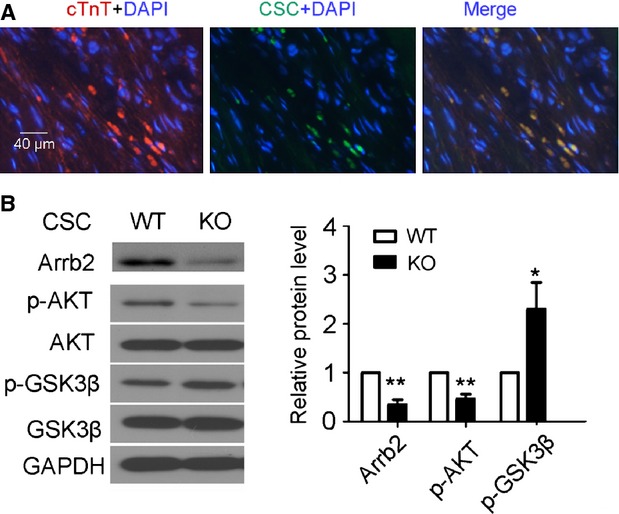
Arrb2/miR-155/GSK3β pathway is important in CSC-mediated cardiac repair. Isolated 2 × 10^5^ Sca-1+ cells from WT or Arrb2-KO mice were injected immediately into infarcted and border zones of the mouse heart after myocardial infarction (MI). Hearts were then reperfused for 1 hr. After 2 weeks, 2-mm sections of hearts near the mid-ventricles were collected. (**A**) Fluoresence microscopy of hearts for WT mice with MI injected with WT Sca-1+ cells and stained with cTnT. Red shows cardiomyocytes; green shows injected Sca-1+ cells; blue shows DAPI-stained cell nuclei; scale bar, 40 μm; *n* = 6. (**B**) Western blot analysis of the expression of Arrb2, total Akt and p-Akt, and total and p-GSK3β. GADPH was a loading control. The column shows the quantification of the protein expression. Protein levels were normalized to GAPDH or total protein; *n* = 3; *P < 0.05; **P < 0.01 *versus*WT.

To determine the role of the Arrb2/miR-155/GSK3β pathway in CSC-mediated cardiac repair *in vivo*, we injected stem cells from WT and KO mice into the hearts of mice with myocardial infarction. After 2 weeks, immunofluorescence assay revealed that the injected Sca-1+ cells could differentiate into myocardial cells (Fig. 6A). Arrb2 protein was expressed in Arrb2-KO mice after transfer of Sca-1+ CSCs from WT mice (Fig. 6B), which suggests that the transplanted cells could survive in the mice with myocardial infarction. Furthermore, in KO mice, the low protein level of Arrb2 caused low phosphorylation of Akt and high phosphorylation of GSK3β, which agrees with *in vitro* results (Fig. 6B).

Cardiac function analysis showed that myocardial infarction (injected with medium) impaired cardiac function significantly as compared with the sham control (*P* < 0.01 *versus* sham), and transplantation of Sca-1+ cells from WT mice could protect cardiac function, including ejection fraction, cardiac output, stroke volume and Vmax (*P* < 0.01 *versus* WT mice injected with medium). However, cardiac function measures did not differ with transplantation of Sca-1+ cells from Arrb2-KO and from WT mice, except for impaired indexes of arterial elastance and Tau-weiss (*P* < 0.01 *versus* WT mice injected with medium; *P* < 0.01 *versus* WT mice injected with WT Sca-1+ CSCs) (Table 1).

**Table 1:** Effects of Sca-1+ CSCs on the cardiac function of wild-type mice with myocardial infarction

	Sham	14 days after myocardial I/R injury		
		
		Vehicle	Wild-type sca-1 cells	Arrb2 KO sca-1 cells
HR (b.p.m)	477 ± 23.3	468 ± 23.6	419 ± 5.6	505 ± 23.9
%EF	69.6 ± 1.5	45.6 ± 1.5*	60.3 ± 2.2*,†	57.3 ± 1.2*,†
LVDP, mmHg	84.4 ± 0.9	71.3 ± 6.3*	62.0 ± 2.2	70.7 ± 5.3
CO (μl/min.)	8410 ± 982.3	3491 ± 448.1*	6123 ± 662.6†	6962 ± 606.5†
E(a)	3.9 ± 0.3	9 ± 1.1*	4.18 ± 0.5†	6.4 ± 0.4†,‡
Tau-weiss	12.2 ± 0.9	8.5 ± 1.1*	11.1 ± 0.3†	7.5 ± 1.3*,‡
EDV	25.2 ± 0.9	18.2 ± 1.3*	24.2 ± 1.3†	22.5 ± 0.7
Dp/dt max	7994 ± 880	5595 ± 1783	4165 ± 75	9055 ± 3066
Dp/dt min	6547 ± 700	5394 ± 2382	4383 ± 393	9203 ± 2129
Stroke volume	17.7 ± 5.9	9 ± 2.2	10.6 ± 5.9	13.7 ± 1.6
Vmax (μl)	26.3 ± 4.0	20.6 ± 3.7	24.6 ± 2.7	23.9 ± 1.9

* *P* < 0.01 *versus* sham.

† *P* < 0.01 *versus* WT mice injected with medium.

‡ *P* < 0.01 *versus* WT mice injected with WT type Sca-1+ cells.

Data are mean ± SEM of six experiments.

HR: heart rate; EF: ejection fraction; LVDP = ESP-EDP; ESP: end-systolic pressure; EDP: end-diastolic pressure; CO: cardiac output; E(a): arterial elastance.

To exclude the affection of background expression of Arrb2 in WT mice, we transplanted WT or Arrb2 KO Sca-1+ CSCs to Arrb2 KO mice with myocardial infarction, high level of Arrb2 equal to better performance of cardiac function, including ejection fraction, cardiac output, stroke volume and Vmax (Table 2), which verified the important role of Arrb2 in heart repair.

**Table 2:** Effects of Sca-1+ CSCs on the cardiac function of Arrb2-KO mice with myocardial infarction

	14 days after myocardial I/R injury			
	
	%EF	CO (μl/min.)	Stroke volume	Vmax (μl)
Wild-type sca-1 cells	67.4 ± 2.3	11493 ± 2600	21.16 ± 1.56	31.35 ± 1.3
Arrb2 KO sca-1 cells	48.95 ± 1.6*	7174 ± 755*	13.29 ± 1.1*	27.25 ± 3.1*

* *P* < 0.05 compared with wild-type sca-1 injection.

Data are mean ± SEM of six experiments.

EF: ejection fraction; CO: cardiac output.

## Discussion

Recently, both experimental and clinical findings have revealed that the heart can replace cardiomyocytes throughout life, but this response is inadequate to compensate for major injuries such as myocardial infarction [18]. So resident CSCs could be stimulated to differentiate into cardiomyocytes. Resident Sca-1+ CSCs, existing in humans and mice [4,19], have therapeutic functions on the heart because of their differentiation potential. We used the 5aza-induced differentiation model *in vitro*, and showed that Arrb2 could promote the differentiation of Sca-1+ cells to cardiomyocytes, which suggested an important role of Arrb2 in Sca-1+ cell transition and promoted us to explore the mechanisms of Arrb2 mediated Sca-1+ CSCs transition to cardiomyocyte. As 5-Azacytidine can induce gene expression through demethylation [20], we deduced that 5-Azacytidine regulated β-arrestin2 expression by decreasing the degree of methylation of the β-arrestin2 gene or other genes. In this study, we focused on the pathway regulated by β-arrestin2, but how 5-Azacytidine regulated β-arrestin2 expression still needs further research.

MiR-155, a well-known multifunctional miRNA, was indicated to play a crucial role in various physiological and pathological processes such as haematopoietic lineage differentiation, immunity, inflammation, cancer, and cardiovascular diseases [21], but its role in CSC differentiation is not clear. Our results showed that miR-155, the predicted regulator of Arrb2, inhibited the 5aza-induced differentiation of Sca-1+ cells to cardiomyocytes and was regulated by Arrb2. So miR-155 might locate downstream of Arrb2. However, dual luciferase reporter assay showed that miR-155 also inhibited the expression of Arrb2. We suggest a loop pathway between miR-155 and Arrb2, which explains the mechanism for its participation in regulating cardiovascular diseases.

As the downstream of Arrb2 and the target of miR-155, GSK3β promoted the 5aza-induced murine Sca-1+ cell differentiation. This result is the same as its role in cardiomyocyte differentiation of murine bone marrow-derived mesenchymal stem cells [22]. Although GSK3β is regulate by Arrb2 in cell apoptosis and target by miR-155 in T-cell proliferation has been reported [5,23], we analysed the relationship among the three factors, and explored the important roles of Arrb2/miR-155/GSK3β pathway in cardiomyocyte differentiation of Sca-1+ cell. Furthermore, we verified that AKT and GSK3β are downstream of β-arresin2. However, GSK3β activity was not affected by Akt phosphorylation as usual. As the protected function of GSK3β to regional myocardial ischaemia/reperfusion injury has been verified [24,25], so the Arrb2/miR-155/GSK3β pathway might be a new target for CSC-mediated cardiac repair.

We transplanted CSCs from WT or Arrb2 KO mice into mice with myocardial infarction to analyse the function of Arrb2 in CSC-participating cardiac repair. In WT infarcted mice, Arrb2–KO CSCs showed the same protective functions, except for arterial elastance perhaps because the background of Arrb2 in WT infarcted mice affected its actual role. Otherwise, it might be caused by the interference of the adrenal-dependent neurohormonal mechanisms. β-arrestins (including Arrb1and Arrb2) have been shown to activate epidermal growth factor receptor by eliciting a G-protein–independent signals *in vitro*, so they might be beneficial for the failing heart. However, with regard to the heart, Arrb1 preferred to perform a G-protein dependent function, and regulates the majority of cardiovascular G protein-coupled receptors, especially adrenal and central sympathetic nervous system α2ARs, to perform a negative impact on post-myocardial infarction heart failure *via* cardiac and adrenal-dependent neurohormonal mechanisms [26]. Arrb2 has the same effect as Arrb1 on cardiac β1ARs and Adrenal α2AR internalization [27]. So it is not strange that Arrb2 also protect the heart from damage. As Arrb2′s role was affected by adrenal-dependent neurohormonal mechanisms, only development of tissue-specific KO mice can provide definitive answers to this important question. However, low Arrb2 level agreed with decreased phosphorylation of AKT and increased phophorylation of GSK3β, findings also found *in vitro*. Furthermore, we transplanted WT or Arrb2 KO Sca-1+ CSCs to Arrb2 KO mice with myocardial infarction, high level of Arrb2 equal to better performance of cardiac function, verified the vital function of Arrb2 in cardiac repair. The Arrb2/miR-155/GSK3β pathway may be a new mechanism with implications for treatment of heart disease.
